# “Non-Classical” Platinum Complexes: A Concise Review

**DOI:** 10.3390/ijms26136270

**Published:** 2025-06-28

**Authors:** Adriana Bakalova, Nina Ruseva, Emiliya Cherneva

**Affiliations:** 1 Department of Chemistry, Faculty of Pharmacy, Medical University of Sofia, 2 Dunav Str., 1000 Sofia, Bulgaria; cherneva@pharmfac.mu-sofia.bg; 2 Department of General and Inorganic Chemistry, University of Chemical Technology and Metallurgy, 8 Kliment Ohridsky Blvd., 1797 Sofia, Bulgaria; nina.ruseva@uctm.edu; 3 Institute of Organic Chemistry with Centre of Phytochemistry, Bulgarian Academy of Sciences, Acad. G. Bonchev Str., Build 9, 1113 Sofia, Bulgaria

**Keywords:** Pt(IV) complexes, *trans*-Pt complexes, mixed ammine/amine platinum complexes, cytotoxic activity

## Abstract

The utilization of platinum complexes in medicine continues to be a prevalent treatment modality for diverse tumour types. However, it should be noted that certain platinum complexes are characterized by a high degree of toxicity. In recent years, there has been a focus among scientists on synthesizing “non-classic” platinum complexes, such as those with a *trans*-configuration, Pt(IV) complexes, and mixed ammine/amine platinum complexes, with the aim of reducing the toxic side effects of certain platinum complexes, including cisplatin. For instance, newly synthesized platinum complexes with a *trans*-configuration exhibited substantial cytotoxic activity which was comparable to that of the corresponding *cis*-isomers and cisplatin. This finding challenged the prevailing *cis*-geometry paradigm and prompted a re-evaluation of the structural activity relationships (SARs) of antitumour platinum complexes. It is widely accepted that Pt(IV) complexes act as prodrugs and release the active Pt(II) species. This property renders them promising candidates as anticancer drugs. Furthermore, it has been established that mixed ammine/amine platinum complexes are less toxic than cisplatin. In addition, compared to cisplatin, they have been observed to have equivalent or greater cytotoxic activity.

## 1. Introduction

Rosenberg’s serendipitous discovery of the antiproliferative activity of cisplatin was a major breakthrough in cancer treatment [1,2,3,4,5]. Cisplatin is a chemotherapeutic agent that has been widely and successfully used to treat various types of cancer, such as solid organ malignancies, including lung, ovarian, testicular, and head and neck cancers [6,7,8]. However, many serious side effects have been reported, including nephrotoxicity, neurotoxicity, ototoxicity, nausea, and vomiting [9,10,11,12,13]. Due to these toxic side effects, scientists have focused on the synthesis and investigation of novel and more efficient platinum complexes.

Carboplatin is a second-generation platinum compound with a structure similar to that of cisplatin. Carboplatin was developed on the assumption that the presence of a more stable leaving group like a 1,1-cyclobutanedicarboxylic acid anion instead of a chloride ion would reduce toxicity without compromising antitumour efficacy [14]. Subsequent research demonstrated the accuracy of this hypothesis. Despite the reduced pharmacological efficacy of carboplatin, a positive aspect of its use is the reduction of systemic toxicity, particularly nephrotoxicity [15,16,17,18]. On the other hand, carboplatin commonly causes myelosuppression [19].

Oxaliplatin, as a third-generation Pt(II) drug, has been authorized for the treatment of metastatic colorectal cancer and has also demonstrated efficacy against lung and ovarian cancer cell lines [14,20]. In comparison with cisplatin, it contains a more stable leaving group and the R,R-diacetylcyclohexane (DACH) chelating ligand [21]. In addition, oxaliplatin demonstrates an alternative resistance profile and generally exhibits superior tolerability compared to cisplatin [22,23,24].

Going further, a plethora of Pt(II) compounds have obtained regulatory approval for utilization. For example, Nedaplatin (in Japan, 1995), Heptaplatin (in Republic of Korea, 1999), Lobaplatin (in China, 2003), Miriplatin (in Japan, 2009), and Dicycloplatin (in China, 2012) [15]. This further enriches the pharmacological armamentarium for cancer treatment.

Recent progress in the field of platinum(IV) prodrugs has been significant. The Pt(IV) complexes are emerging as an alternative class of platinum-based drugs, with the aim of minimizing side effects, improving drug efficiency, and overcoming drug resistance [5].

In recent decades, it has been acknowledged that substituting NH_3_ with alternative amino ligands (L or L′) in the *trans*-position of the following structure (*trans*-[PtCl_2_(L)(L′)]) can enhance the cytotoxic properties of the compounds [25].

Following the indication in the preliminary research findings that mixed ammine/amine platinum complexes were less toxic than the cisplatin, the scientific community embarked upon synthesizing and studying these complexes [26].

The focus of this review is a range of “non-classical”, potentially cytotoxic, platinum complexes. These complexes include Pt(IV) compounds, *trans*-platinum compounds and mixed ammine/amine Pt(II) compounds.

## 2. “Non-Classical” Platinum Complexes with Cytotoxic Activity

### 2.1. Pt(IV) Complexes

**Figure 1:**
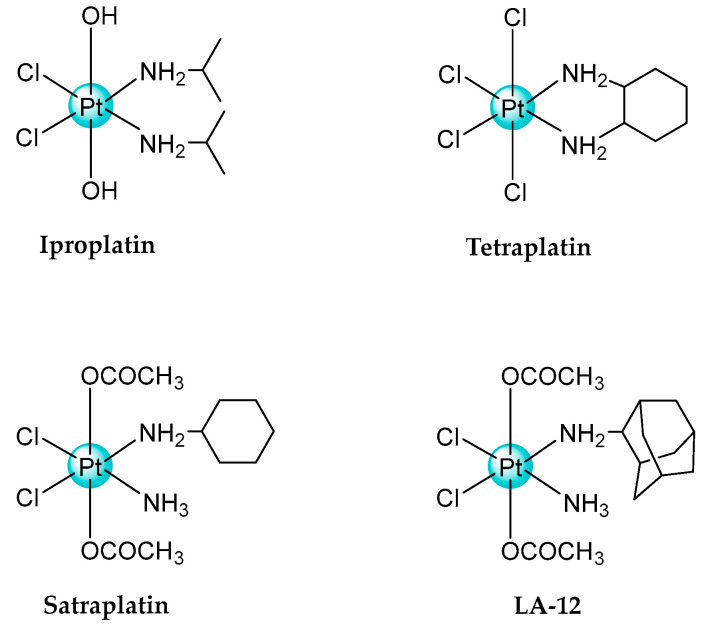
Chemical structures of Pt(IV) complexes in clinical uses.

In order to overcome the vast number of serious side effects of cisplatin and other Pt(II) complexes, intensive research has led to the development of new Pt(IV) complexes. The utilization of platinum(IV) complexes as metal-based cancer prodrugs has emerged as a highly promising area of research [27]. Indeed, a small number of Pt(IV) complexes have reached clinical trials over the years. For instance, iproplatin, otherwise known as *cis*,*trans*,*cis*-dichloridodihydroxido-bis(isopropylamine)platinum(IV), was tested in phase II and III trials (Figure 1). Nevertheless, it was ultimately rejected on the basis that it exhibited reduced antitumour activity in comparison to cisplatin and carboplatin [15]. Ormaplatin or tetraplatin, [tetrachlorido(1,2-diaminocyclohexane)platinum(IV)], entered phase I studies but was also rejected as more neurotoxic than cisplatin [28,29]. A compound that is undergoing phase I/II clinical trials is LA-12, or ((OC-6-43)-bis(acetato)(1-adamanthylamine) aminedichloridoplatinum(IV)). Unfortunately, no data are available yet [30]. To date, only satraplatin, or *cis*,*trans*,*cis*-dichlorido-bis(acetato)amminecyclohexylamineplatinum(IV), has successfully passed phase III clinical trials, demonstrating anticancer activity in men with metastatic castration-resistant prostate cancer [31,32]. 

Nevertheless, as there has been no improvement in overall survival, further clinical trials are currently underway.

The Pt(IV) complexes have an octahedral geometry [33]. The coordination sphere is saturated and leads to stability of the ligand substitution reactions, preventing their inactivation by biomolecules present in the human body [32].

Pt(IV) complexes are thought to be more inert than their Pt(II) counterparts, which accounts for the slower ligand substitution.

As was established at the time of Rosenberg’s discovery of cisplatin, platinum(IV) complexes, for instance, *cis*-[PtCl_4_(NH_3_)_2_], possess anticancer properties [1].

In relation to synthesis and the method of administration of drugs into the body, platinum(IV) complexes demonstrate several advantages in comparison to platinum(II) complexes [34].

- Firstly, it is evident that the kinetic inertness of platinum(IV) complexes reduces the probability of adverse reactions in vivo [35]. This phenomenon can be attributed to the increased stability of these complexes exhibit in acidic environments formed as a consequence of bacterial activity (specifically, the synthesis of folic acid for DNA). Consequently, the complexes demonstrate heightened effectiveness against these bacterial cultures [36].
- Secondly, the capacity of the compounds to penetrate cells and reduce themselves inside the cell is crucial. It has been established that the reduction of Pt(IV) to Pt(II) by biological agents is a prerequisite for their antitumour activity [29,37].
- Finally, Pt(IV) complexes have been demonstrated to retain their efficacy against cancer cells in hypoxic conditions, thus establishing them as promising candidates for the treatment of avascular tumours [38].

One of the key advantages of Pt(IV) complexes is the ability to modify the axial position of the ligands. This modification allows for the following benefits [39]:

- Greater selectivity of the complexes towards tumour cells;
- Improved cellular uptake;
- Greater tolerance of the complexes in biological media.

Coordination sites located in the axial position have been shown to be capable of binding other biologically active molecules [40,41,42,43,44]. It was determined that these complexes could be regarded as a novel class of Pt(IV) complexes, comprising two biologically active components within a single molecule, and thus hold particular significance [45,46,47].

In recent times, scientific interest has been directed towards the utilisation of cisplatin, oxaliplatin, or other Pt(II) complexes in the synthesis of novel Pt(IV) complexes, accompanied by diverse axial ligands.

**Figure 2:**
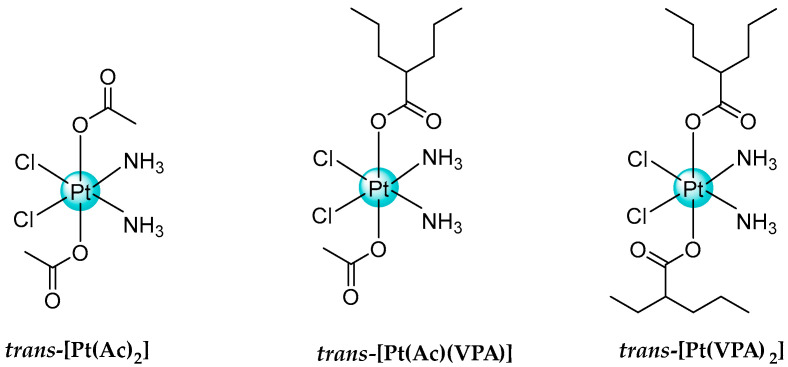
Possible chemical formulas of *cis*,*trans*,*cis*-[Pt(NH_3_)_2_(AcO)_2_Cl_2_], *cis*,*trans*,*cis*-[Pt(NH_3_)_2_(AcO)(VPA)Cl_2_], and *cis*,*trans*,*cis*-[Pt(NH_3_)_2_(VPA)_2_Cl_2_].

For instance, Novohradsky et al. in 2015 synthesized three new Pt(IV) complexes with the chemical formulas *cis*,*trans*,*cis*-[Pt(NH_3_)_2_(AcO)_2_Cl_2_], *cis*,*trans*,*cis*-[Pt(NH_3_)_2_(AcO)(VPA)Cl_2_], and *cis*,*trans*,*cis*-[Pt(NH_3_)_2_(VPA)_2_Cl_2_], where OAc is acetate and VPA is valproic ions (Figure 2) [48].

The newly obtained complexes were analogues of cisplatin and were characterized by NMR spectral analysis. The complexes were evaluated for antiproliferative activity on A2780 and A2780cisR human ovarian carcinoma cell lines. The cytotoxic activity of platinum(IV) derivatives of cisplatin with axial valproic acid (VPA) ligands is significantly superior to that of conventional cisplatin. It has been demonstrated that the Pt(IV) complex with two acetate ions in the axial position exhibits reduced cytotoxicity in comparison to that of cisplatin [48].

A series of ten novel mono- and bis-carboxylated Pt(IV) complexes on a cisplatin scaffold with indole-3-acetic acid (IAA) and indole-3-propionic acid (IPA) as biologically active axial ligands were synthesized and characterized [49].

**Figure 3:**
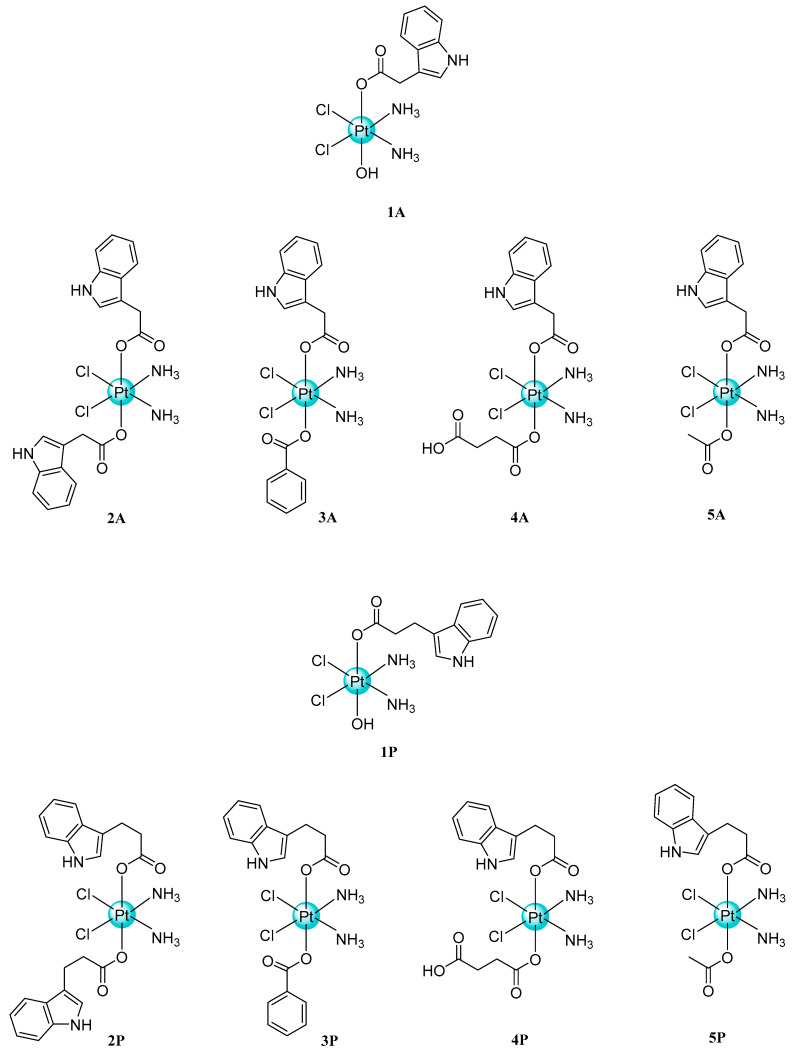
Chemical formulas of novel mono- and bis-carboxylated Pt(IV) complexes.

Indole compounds have been shown to have antioxidant and radical scavenging properties [49]. Indole-3-propionic acid has been found to reduce auto-oxidation and protect against lipid oxidation [50,51]. In complexes **(1A)** to **(5A)** (Figure 3), one axial ligand is indole-3-acetic acid and the other is OH^−^, IAA, or three different anhydrides (benzoic, acetic, and succinic anhydrides). Complexes **(1P)** to **(5P)** contain indole-3-propionic acid as one axial ligand, with the other axial ligand being once again OH^−^, IAA, and three different anhydrides (benzoic, acetic, and succinic acids).

The complexes were studied for cytotoxic activity on a panel of human tumour cell lines such as pancreatic (BxPC3), colorectal (HCT-15), breast (MCF-7), cervical (A431), lung (A549), and melanoma (A375) cancers. The results obtained were compared with those of the metal-free ligands and the reference drug, cisplatin. The Pt(IV) complex with a hydroxide ion in the axial position and indole-3-propionic acid as the second axial ligand **(1P)** demonstrated significant antiproliferative activity and was approximately three times more effective than cisplatin. Moreover, the unsymmetrical Pt(IV) complex with benzoic acid instead of a hydroxide ion exhibited cytotoxicity that was analogous to that of the **(1P)** derivative [49]. The introduction of an additional carboxyl ligand (benzoate, acetate, or succinate) in the axial position resulted in the formation of Pt(IV) derivatives. The activity profile of these derivatives was found to be contingent on the nature of the substituent.

**Figure 4:**
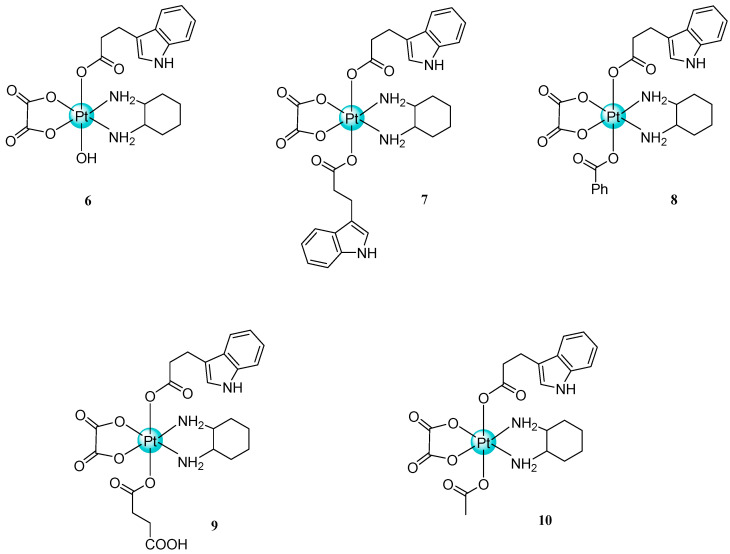
Chemical structures of Pt(IV) complexes based on an oxaliplatin and indole-3-propionate.

In addition, five novel Pt(IV) complexes were synthesized by the same scientists, based on an oxaliplatin scaffold as opposed to cisplatin and indole-3-propionate (IPA) [52]. The chemical formulas of the complexes are as follows: *trans*-[Pt(DACH)(OXA)(IPA)(OH)] **(6)**, *trans*-[Pt(DACH)(OXA)(IPA)_2_] **(7)**, *trans*-[Pt(DACH)(OXA)(IPA)(bz)] **(8)**, *trans*-[Pt(DACH)(OXA)(IPA)(suc)] **(9)**, and *trans*-[Pt(DACH)(OXA)(IPA)(AcO)] **(10)**, where DACH is 1,2-diaminocyclohexane (1R,2R)-(-), OXA = oxalate, IPA = indole-3-propionate, bz = benzoate, suc = succinate, and AcO = acetate ions (Figure 4).

The characterization of the complexes was conducted through a range of analytical methods, including elemental analyses, IR, NMR spectroscopy, and mass spectrometry. The newly synthesized platinum(IV) complexes were then subjected to estimation in terms of their cytotoxic activity. The evaluation was conducted using two human ovarian carcinoma cell lines: the cisplatin-sensitive A2780 cell line and the cisplatin-resistant A2780cis cell line. In comparison with cisplatin, the complexes exhibit reduced cytotoxicity, particularly in the A2780 cell line exhibiting sensitivity. Conversely, the complexes demonstrate augmented cytotoxic activity in comparison with the reference drug oxaliplatin. The *cis*,*trans*,*cis*-[Pt(DACH)(IPA)(OH)(OXA)] complex has been identified as the most cytotoxic active of the other four complexes and also of oxaliplatin on ovarian cancer cells (A2780). Compared to previously reported cisplatin-based analogues, Pt(IV) complexes based on an oxaliplatin scaffold demonstrate only moderate levels of cellular toxicity [49]. This discovery is consistent with the observations reported in the literature concerning other oxaliplatin/cisplatin prodrugs [53]. The relatively slow reduction kinetics of Pt(IV) derivatives of oxaliplatin can be identified as the underlying cause of this phenomenon.

After a twelve-month period, Papadia et al. synthesized and characterized six new Pt(IV) complexes. They were based on an oxaliplatin analogue containing *trans*-1,2-diamino-4-cyclohexene (DACHEX) instead of 1,2-diaminocyclohexane (DACH), and hydroxo, acetate, and benzoate groups as axial ligands [54].

**Figure 5:**
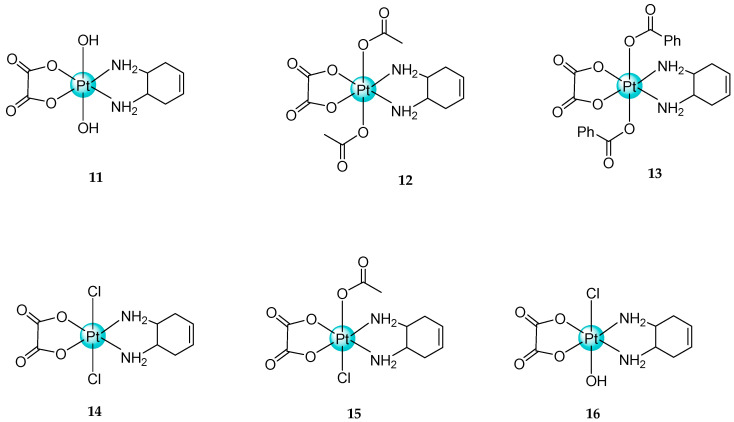
Chemical formulas of Pt(IV) complexes, synthesized on the basis of oxaliplatin and DACHEX.

The chemical formulas are: *cis*,*trans*,*cis*-[Pt(OXA)(OH)_2_(DACHEX)] **(11)**, *cis*,*trans*,*cis*-[Pt(OXA)(AcO)_2_(DACHEX)] **(12)**, *cis*,*trans*,*cis*-[Pt(OXA)(BzO)_2_(DACHEX)] **(13)**, *cis*,*trans*,*cis*-[Pt(OXA)Cl_2_(DACHEX)] **(14)**, *cis*,*trans*,*cis*-[Pt(OXA)(AcO)Cl(DACHEX)] **(15)**, and *cis*,*trans*,*cis*-[Pt(OXA)(OH)Cl(DACHEX)] **(16)**, where AcO is acetate, BzO is benzoate, and OXA is oxalate (Figure 5). The Pt(IV) complexes were synthesized using oxaliplatin as the starting Pt(II) complex. The novel complexes were characterized by ^1^H and ^13^C NMR and ESI-MS spectra.

The complexes were evaluated for cytotoxicity on a panel of human tumour cell lines, such as pancreatic (PSN-1), colon (HCT-15 and LoVo), cervical (A431), and ovarian cancer (2008). The reference compounds employed in this study were cisplatin and oxaliplatin. The new Pt(IV) complexes exhibited remarkable antitumor activity, which was found to be significantly higher than that of cisplatin and oxaliplatin on the cell lines examined. It was established that complex **(13)**, with benzoate axial ligands, which serve to increase its lipophilicity, was active even at nanomolar concentrations.

**Figure 6:**
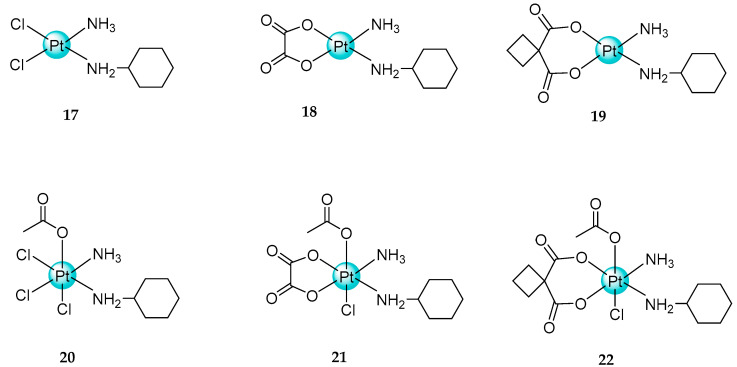
Pt(II) and Pt(IV) complexes with cyclohexylamine as a carrier ligand and chloride, 1,1-cyclobutanedicarboxylate, and oxalate anions as leaving groups.

Three platinum(II) complexes with asymmetric carrier ligands and three platinum(IV) complexes with lipophilic ligands in their axial positions have been designed and synthesized [55]. The Pt(II) complexes have cyclohexylamine as a carrier ligand and chloride, 1,1-cyclobutanedicarboxylate, and oxalate anions as leaving groups (Figure 6 **(17**–**19)** complexes). It has been determined that the Pt(IV) complexes have identical ligands in equatorial positions as well as Pt(II) complexes. Furthermore, in the axial positions the ligands are found to be the same in all three Pt(IV) complexes (Figure 6, **(20**–**22)** complexes).

A comparative study of the cytotoxicity of Pt(II) and Pt(IV) complexes was conducted on a series of human carcinoma cell lines, including human hepatocellular carcinoma (SMMC-7721), human breast cancer (MCF-7), human lung cancer (A549), and human colorectal cancer (SW480). The evaluation was accomplished by means of the MTT assay. In vitro results showed that the new platinum(IV) complexes, except **(19)** and **(22)**, displayed strong cytotoxicity toward four human cancer cell lines. Compound **(17)** was twofold to sevenfold more effective than cisplatin against four human cancer cell lines. Compound **(18)** was roughly six and five times more effective than cisplatin against MCF-7 and SW480 cells, respectively. The improved and selective cytotoxicity of **(18)** towards different cancer cells could be due to the use of dicarboxylate ions as leaving groups. This gives the compound lower reduction potentials and slower reduction rates than its chloride analogues **(17)** [56]. Compound **(20)** was more effective than satraplatin and cisplatin against the tested cancer cell lines, except for SMMC-7721. The chloride ions in the equatorial positions of **(17)** and **(20)** would leave the compounds quickly due to the trans effect and low chloride ion concentration in the cells. This would allow the Pt ion to easily bind to the guanine in DNA, which would which increase the Pt ion’s DNA-binding ability and thus improve its anticancer efficacy [57]. It was discovered that compound **(21)** was the most cytotoxic derivative of satraplatin. It was found that the IC_50_ values of compound **(21)** were two- to eightfold lower than those of satraplatin and cisplatin in four human cancer cell lines. The reduction of the platinum(IV) analogues to the platinum(II) congener appears to be necessary for binding to DNA. Compared with compounds **(17)** and **(18)**, the axial carboxylato ligands and chloride ligands in compounds **(20)** and **(21)** featuring intermediate reduction potentials were supposed to fit best into the biological activity window [58]. Moreover, compound **(21)** with dicarboxylato equatorial ligands may exhibit lower reduction potentials and slower reduction rates than analogous compound **(20)** with dichlorido equatorial ligands [59]. Because of its favourable chemical structure, compound **(21)**, as a novel ammine/amine platinum(IV) dicarboxylate, is expected to be an orally administered platinum candidate [60,61].

**Figure 7:**
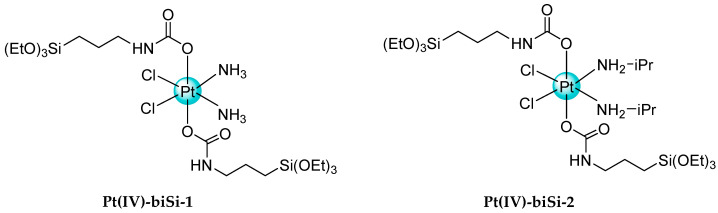
*Cis*-dichlorido(diamine)-*trans*-[3-(triethoxysilyl)propylcarbamate]platinum(IV) (Pt(IV)-biSi-1) and *cis*-dichlorido(diisopropylamine)-*trans*-[3-(triethoxysilyl) propyl carbamate]platinum(IV) (Pt(IV)-biSi-2).

A novel approach to the design of new Pt(IV) complexes involves the incorporation of various ligands in different positions, while maintaining the pharmacological activity of Pt(II) complexes. Thus, the synthesis of two Pt(IV) complexes with aliphatic amines and chloride ligands in the equatorial plane, along with two bis-organosilane moieties in axial positions, *cis*-dichloro(diamine)-*trans*-[3-(triethoxysilyl)propylcarbamate]platinum(IV) (Pt(IV)-biSi-1) and *cis*-dichloro(diisopropylamine)-*trans*-[3-(triethoxysilyl) propyl carbamate]platinum(IV) (Pt(IV)-biSi-2), was described (Figure 7) [56]. The complexes were evaluated for cytotoxic activity in vitro against two human colorectal adenocarcinoma cell lines (HCT 116 and HT-29) and a non-tumourigenic intestinal epithelial crypt cell line (HIEC6). The Pt(IV)-biSi-2 complex demonstrated a higher level of antiproliferative activity against colorectal tumour cell lines, while exhibiting a reduced degree of toxicity against control and non-tumourigenic intestinal cells.

**Figure 8:**
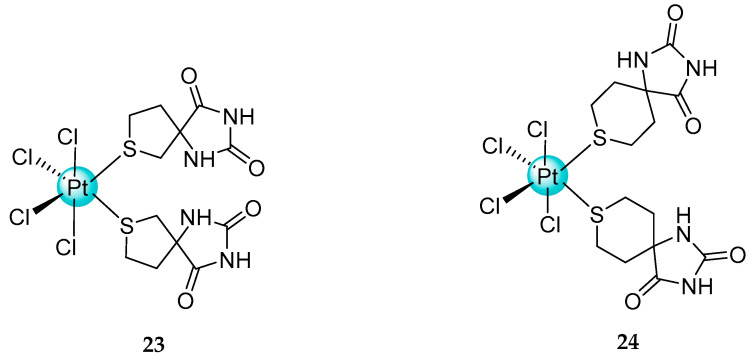
Chemical formulas of the Pt(IV) complexes with 3-thiolanespiro-5′-hydantoin (L_1_) **(23)** and 4-thio-1H-tetrahydropyran-5′-spiro-hydantoin (L_2_) **(24)**.

In 2014, Bakalova and colleagues synthesized two new Pt(IV) complexes with 3-thiolanespiro-5′-hydantoin (L_1_) and 4-thio-1H-tetrahydropyran-5′-spiro-hydantoin (L_2_) (Figure 8). Their general formulae were established as *cis*-[Pt(L_1_,_2_)_2_Cl_4_] [62]. The complexes were subjected to in vitro testing for their potential anticancer properties using four human tumour cell lines (SKW-3, HL-60, EJ, and LAMA-84). The results demonstrated that the compounds exhibited higher levels of anticancer activity in comparison to their Pt(II) analogues with the general formula *cis*-[Pt(L_1,2_)_2_Cl_2_] with the same ligands against the used tumour cell lines. 

**Figure 9:**
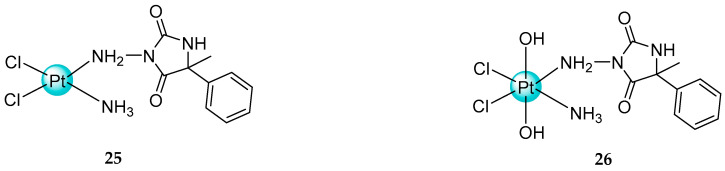
Molecular formulas of the Pt(II) and Pt(IV) complexes with 3-amino-5-methyl-5-phenyl-hydantoin.

Cherneva et al. synthesized and investigated two novel Pt(II) and Pt(IV) complexes with 3-amino-5-methyl-5-phenyl-hydantoin in 2017 (Figure 9) [63]. In order to establish the geometry of the complexes, a DFT method was employed that utilized the B3LYP with the LANL2DZ basis set. It was demonstrated that the platinum ion exhibits monodentate coordination with a nitrogen atom from the NH_2_ group of the hydantoin ring. The compounds were evaluated for in vitro cytotoxicity on human tumour cell lines such as HT-29, MDA-MB-231, and HL-60. A novel Pt(IV) complex with OH^−^ ligands in the axial position and the formula *cis*,*trans*,*cis*-[Pt(NH_3_)L(OH)_2_Cl_2_] exhibits higher antiproliferative activity than the *cis*-[Pt(NH_3_)(L)Cl_2_] complex with the same ligand and reference drug cisplatin on an colon adenocarcinoma cell line (HT-29).

**Figure 10:**
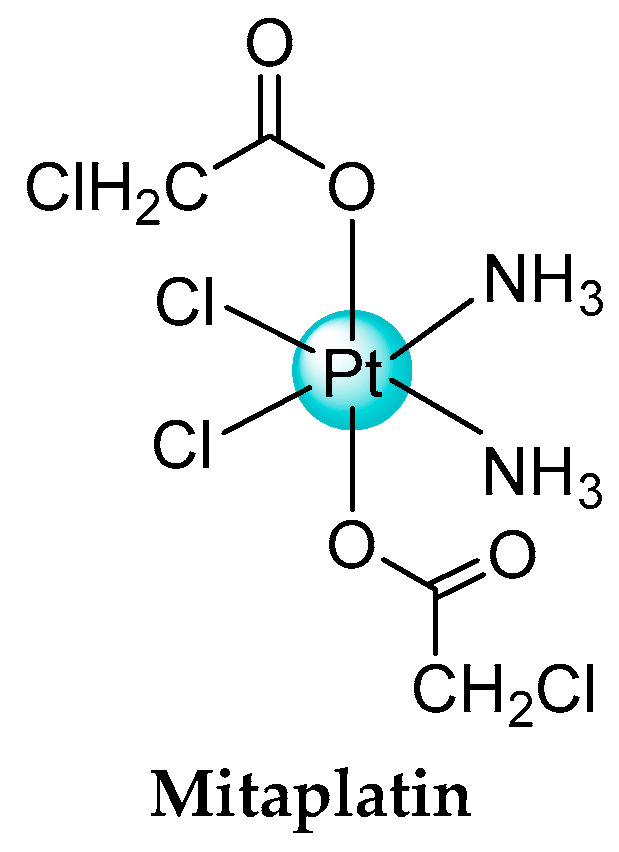
Chemical formula of mitaplatin.

In 2009, Dhar and Lippard published their findings on the first Pt(IV) compound with two dichloroacetate (DCA) ligands directly coordinated to the platinum ion at axial positions [42]. The compound was named “mitaplatin” (OC-6-33)diamminedichlorido-bis-(dichloroacetato)platinum(IV)), where two chloride ions and two ammine groups are located at the equatorial plane (Figure 10).

Mitaplatin has been the subject of extensive research in relation to its cytotoxicity on a panel of human tumour cell lines. They include malignant pluripotent embryonal carcinoma (NTera-2), cervical adenocarcinoma (HeLa), osteosarcoma (U-2OS), lung carcinoma (A549), breast adenocarcinoma (MCF-7), and ovarian endometrial adenocarcinoma (A2780) and its cisplatin-resistant subline (A2780/CP70). The results demonstrated that mitaplatin exhibited comparable and even higher cytotoxicity than cisplatin, particularly in the resistant cell lines [42]. The findings suggest that mitaplatin also exhibits a double-killing mode that may be effective only in cancer cells.

**Figure 11:**
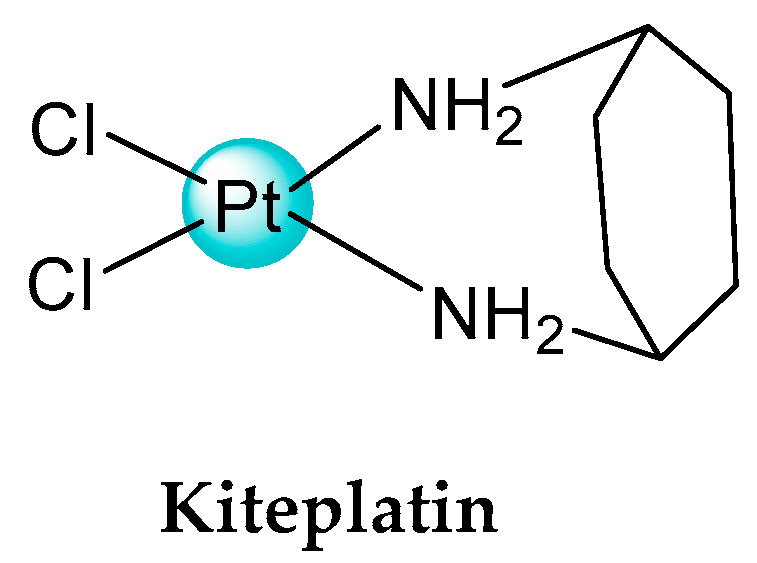
Chemical formula of kiteplatin.

However, it has been demonstrated that not only axial but also equatorial ligands have the capacity to improve the cytotoxicity of Pt(IV) prodrugs [64]. Indeed, the nature of the active Pt(II) complex released following intracellular reduction can be ascribed to the presence of equatorial ligands. In this context, it is noteworthy to mention the Pt(II) derivative [PtCl_2_(*cis*-1,4-DACH)] (DACH = diaminocyclohexane), also designated as kiteplatin, which has recently showed a remarkable antiproliferative activity (Figure 11).

Kiteplatin has been presented to contain an isomeric form of the diamine ligand (1R,2R-DACH) present in oxaliplatin and two chloride ions instead of a dianion of oxalic acid [59]. In vitro studies have indicated that kiteplatin is active against cisplatin-resistant (ovarian C13*) and oxaliplatin-resistant (colon LoVo-OXP) cell lines. This suggests that the activity of kiteplatin may differ from that of cisplatin and oxaliplatin. Moreover, tested in vivo against murine Lewis lung carcinoma, kiteplatin was found to possess an efficacy similar to that of cisplatin and to be better tolerated than the reference metallo-drug [65].

**Figure 12:**
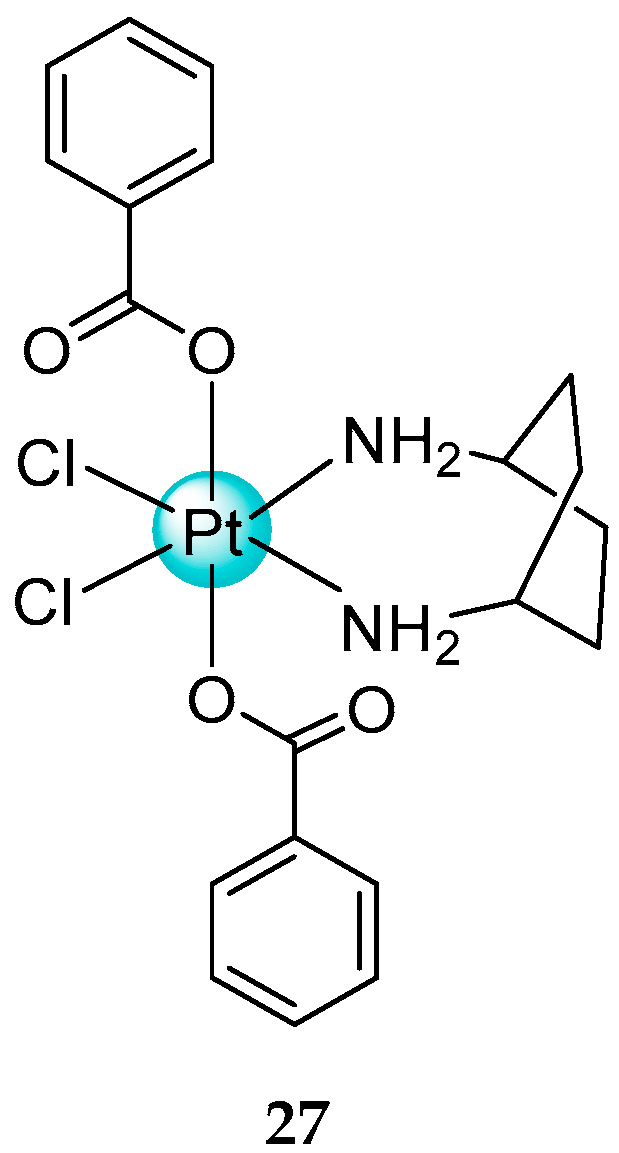
*cis*,*trans*,*cis*-[PtCl_2_(OBz)_2_(*cis*-1,4-DACH)].

In an effort to enhance the activity of kiteplatin, a significant number of researchers have synthesized a variety of platinum(IV) complexes by incorporating two benzoate groups in axial positions [66]. For instance, in 2022, Barbanente et al. prepared a new Pt(IV) complex, *cis*,*trans*,*cis*-[Pt(*cis*-1,4-DACH)(OBz)_2_Cl_2_](OBz = benzoate) **(27)**, and screened it for cytotoxic activity against a panel of human tumour cell lines (Figure 12) [66].

The newly synthesized platinum complex exhibited significantly higher in vitro activity compared to its Pt(II) precursor, prompting the authors to investigate its potential in vivo as well. Further in vitro and in vivo neurotoxicity studies may confirm the compound’s potential as a future antitumour drug for oral administration.

**Figure 13:**
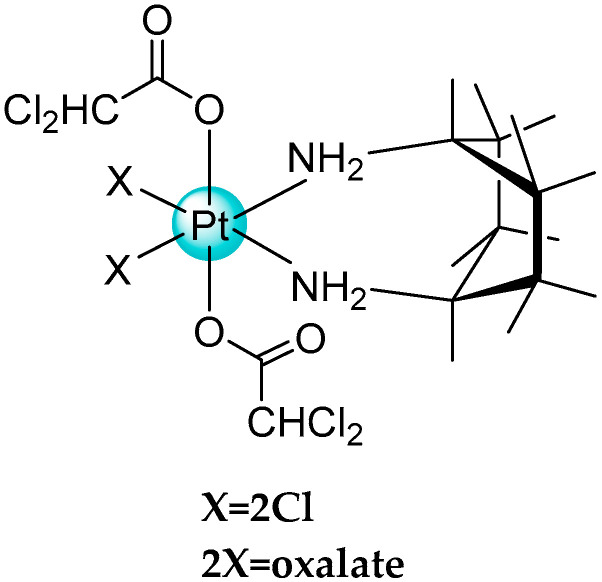
*cis*,*trans*,*cis*-[PtX_2_(DCA)_2_(*cis*-1,4-DACH)].

Furthermore, in 2018, Savino et al. also synthesized the following kiteplatin derivatives: *cis*,*trans*,*cis*-[PtCl_2_(DCA)_2_(*cis*-1,4-DACH)] and *cis*,*trans*,*cis*-[Pt(OXA)_2_(DCA)_2_ (*cis*-1,4-DACH)], where OXA is oxalate ion, DCA is dichloroacetate anion, and *cis*-1,4-DACH is 1,4-diaminocyclohexane. In the complexes, dichloroacetate ions are in the axial position (Figure 13) [64].

The compound *cis*,*trans*,*cis*-[PtCl_2_(DCA)_2_(*cis*-1,4-DACH)] has been shown to be cytotoxic against human pancreatic carcinoma cells and to exhibit high levels of cytotoxicity against neoplastic cells. It can be hypothesized that the use of oxalate ion as a leaving ligand in place of chloride anions may result in a reduction of the cytotoxic activity; it is of note that the reduction potential of Pt(IV) complexes is of great importance to their practical application [67,68]. It has been determined that when complexes of platinum(IV) are susceptible to reduction, the consequence may be the occurrence of serious side effects. Conversely, compounds that are very difficult to reduce may be eliminated from the body without exerting any therapeutic effect. Therefore, by altering the ligands surrounding the complexing agent, the reduction potential of Pt(IV) complexes can be modulated. The primary factors contributing to the stability of Pt(IV) complexes are the ligands, which are situated in the axial position. In this position, hydroxide anions have been found to provide the greatest stability to Pt(IV) complexes, with acetate ions ranking second. Meanwhile, chloride ions exhibited a weak stabilizing effect.

Nevertheless, the reduction rate of Pt(IV) complexes to their Pt(II) counterparts has not been shown to always correspond with the reduction potential [49]. For instance, it has been determined that hydroxide and chloride ligands located in the axial position can act as electron bridges, thereby accelerating the rate of electron transfer from ascorbic acid or thiol to the Pt ion. In contrast, Pt(IV) complexes with carboxylate ions serve as axial ligands and undergo slow reduction due to the weaker electron transfer properties of carboxylate anions [59,69,70,71]. The available data concerning products obtained, reducing agents, and the rate of reduction in vivo is insufficient. In 2007, Nemirovski et al. demonstrated that the in vivo reduction of Pt(IV) complexes to their Pt(II) species was predominantly facilitated by biomolecules as opposed to ascorbic acid or analogous reducing agents [72].

In conclusion, four mechanisms can be identified through which the reduction of Pt(IV) to Pt(II) species through reducing agent (RA) or monoanionic ascorbate AscH^−^ is achieved [73]:

(a) Ligand-bridge electron transfer is a process in which axial ligands form a bridge between the Pt(IV) centre and the (RA). This process facilitates electron transfer and the release of the second ligand in the axial position. It can thus be concluded that the active square planar Pt(II) species are formed.
(b) Ligand-bridge-H transfer is a process characterized by the transfer of two electrons as a result of a shift in a hydride unit (H) from the RA to the bridging ligand. This process leads to the formation of the RA oxidized form and the loss of the axial ligand in the trans-position.
(c) Enolate b-carbon attack is a process that involves the nucleophilic attack of the enolate b-carbon of AscH on one axial ligand (X). Consequently, the formation of the new C-X bond is observed, accompanied by the release of the second axial ligand.
(d) Pt(II)-catalyzed mechanisms are where the reduction of Pt(IV) complex is realized by another Pt(II) complex, the formation of AscH–PtII–X–PtIV–Y dimer, where X and Y are the Pt(IV) axial ligands and the bridging ligand mediates the two electron transfers.

**Figure 14:**
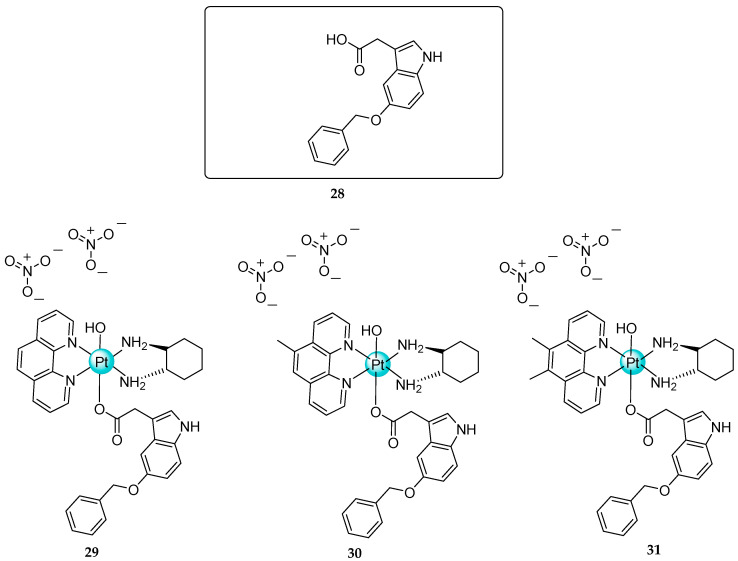
Molecular formulas of compounds **28**–**31**.

Three new Pt(IV) complexes of type [Pt^IV^(H_L_)(A^L^)(X)(OH)]^2+^, where X is the indole-based axial ligand, (**28**), [Pt^IV^(phen)(*SS*-DACH)(5B3A)(OH)]^2+^ ([PHEN*SS*(IV)(5B3A)(OH)]^2+^ (**29**), [Pt^IV^(5-Mephen)(*SS*-DACH)(5B3A)(OH)]^2+^ ([5ME*SS*(IV)(5B3A)(OH)]^2+^ (**30**), and [Pt^IV^(5,6-Me_2_phen)(*SS*-DACH)(5B3A)(OH)]^2+^ ([5,6ME*SS*(IV)(5B3A)(OH)]^2+^ (**31**), were synthesized and investigated (Figure 14) [74].

The purity and chemical formulas of the new complexes were confirmed by elemental analyses, HPLC, NMR, UV, and circular dichroism (CD). The reduction behaviour of the complexes was monitored by NMR at 37 °C in the presence of phosphate-buffered saline (PBS) and ascorbic acid (AsA). The results indicate the ability of the complexes to reduce to the equivalent Pt(II) complexes. Consequently, it is proposed that they can be defined as prodrugs. The complexes were evaluated in vitro for cytotoxicity on panel of human tumour cell lines such as colon (HT29), glioblastoma (U87), breast (MCF-7), ovarian (A2780), lung (H460), skin (A431), prostate (Du145), neuroblastoma (BE2-C), glioblastoma (SJ-G2), pancreas (MIA), the cisplatin-resistant ovarian variant (ADDP), and the non-tumour-derived breast line (MCF10A). Results showed that in most cell lines tested, the complexes are significantly more cytotoxic active than cisplatin, oxaliplatin, and carboplatin.

### 2.2. Trans-Platinum Complexes

Recent years have seen a significant increase in the research focus on *trans*-platinum complexes in the context of the search for new metal drugs [75,76]. According to early classical “Structure–Activity Relationship” (“SAR”), only platinum complexes with *cis*-geometry possess antiproliferative activity [14]. *Trans*-diaminedichloridoplatinum(II) [transplatin], a *trans*-analogue of cisplatin, and other *trans*-platinum complexes are inactive as antitumor drugs [77]. This is most likely due to their kinetic instability and lack of ability to induce structural disruptions in DNA [78].

It is imperative to emphasize that no official registration of any *trans*-Pt(II) and Pt(IV) complexes as anticancer drugs has been conducted to date.

**Figure 15:**
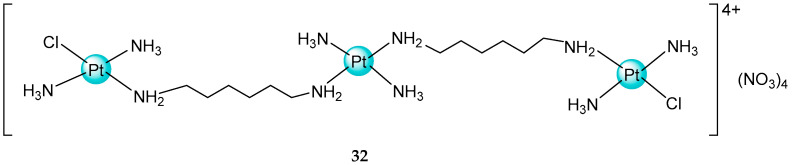
Molecular formula of triplatin tetranitrate.

The only promising polinuclear triplatin tetranitrate complex with a *trans*-configuration (Figure 15) was entered into clinical trials. However, further tests have since been discontinued [15,79,80].

In order to surmount the potential disadvantages and minimize the severe adverse side effects, drug resistance, poor selectivity, and high toxicity of cisplatin, a considerable number of *trans*-platinum(II) complexes have been synthesized and investigated [81]. The complexes showed significant cytotoxic activity comparable to that of the corresponding *cis*-isomers and cisplatin thus breaking the *cis*-geometry paradigm and urging for a re-evaluation of the “SAR” of antitumor platinum complexes [82].

**Scheme 1:**
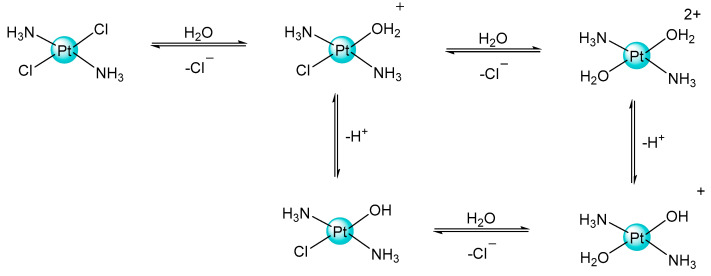
Hydrolytic activation of transplatin.

It has been established that both transplatin and cisplatin are electroneutral complexes. A comparison of the mechanism of action of transplatin and cisplatin reveals that in order to react with DNA, transplatin must first be activated to a mono- and diaqua-complex (Scheme 1) [83].

The removal of the first chloride ion from *trans*-[PtCl_2_(NH_3_)_2_] is relatively easy due to the *trans*-effect of Cl^−^, while the replacement of the second Cl^−^ with H_2_O molecules is not so easy since it is already in a *trans*-position relative to the weaker water molecule [84].

In 1989, Farrell et al. [85] reported the first *trans*-[PtCl_2_L_2_], where L is pyridine and 4-methylpyridine, and it was found to be significantly more cytotoxic than their corresponding *cis*-isomer. This phenomenon may be explained by the distinct mechanisms of action involved. The predominant DNA adducts of *trans*-DDP are 1,3 intrastrand and interstrand cross-links, whereas *cis*-DDP forms 1,2 intrastrand cross-links on DNA [70].

This phenomenon may be attributed to the distinct mechanisms of action involved. The predominant DNA adducts of *trans*-DDP are 1,3 intrastrand and interstrand cross-links, while *cis*-DDP forms 1,2 intrastrand cross-links on DNA.

The replacement of the carrier ligand ammine with a planar aromatic N-donor base, such as pyridine in transplatin, has been demonstrated to result in increased cytotoxicity in cisplatin-sensitive and -resistant cell lines when compared to transplatin [86,87,88,89].

**Figure 16:**
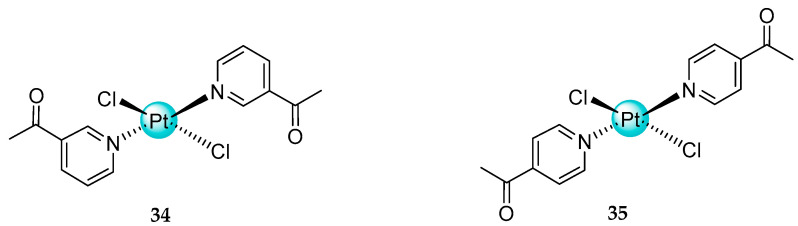
*Trans*-[PtCl_2_(3-acetylpyridine)_2_] **(34**) and *trans*-[PtCl_2_(4-acetylpyridine)_2_] **(35)**.

In 2009, Rakić and co-workers synthesized two new *trans*-[PtCl_2_(n-acetylpyridine)_2_], where n is 3 and 4 (Figure 16) [90].

The compounds were evaluated for antiproliferative activity on nine malignant cell lines: human cervical cancer (HeLa), human osteosarcoma (U2OS), human cisplatin-resistant osteosarcoma (U2OScisR), murine melanoma (B16), human breast cancer (MDA-453, MDA-361, and MCF-7), human colon cancer (LS-174), and human melanoma (FemX). The data revealed that they exhibited moderate to strong cytotoxic activity against the majority of the cell lines tested, but it was different for the two complexes. However, this was not the case for the two complexes. For example, the complex *trans*-[PtCl_2_(4-acetylpyridine)_2_] exhibits cytotoxic activity at low micromolar concentrations. The study revealed that the compound demonstrated the highest level of toxicity towards HeLa cells, which was comparable to that of cisplatin. Furthermore, it was determined that the position of the acetyl group on the pyridine ring was the primary factor contributing to the variation in activity across the range of cell lines examined. Later, the mechanism underlying the in vitro cytotoxic activity of the *trans*-Pt(II) compounds was investigated to understand possible links to their structural differences, such as the position of the acetyl substituent on the pyridine ligand [91].

**Figure 17:**
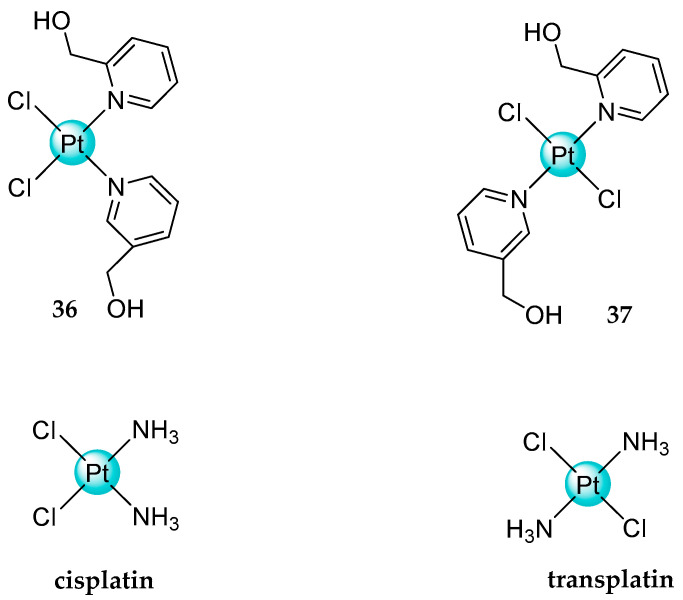
*Cis*-[PtCl_2_(3-hmpy)_2_] **(36)** and *trans*-[PtCl_2_(3-hmpy)_2_] **(37)**, cisplatin and transplatin.

The synthesis of the two additional complexes, *cis*-[PtCl_2_(3-hmpy)_2_] **(36)** and *trans*-[PtCl_2_(3-hmpy)_2_] **(37)**, where hmpy denotes 3-hydroxymethylpyridine, occurred at a subsequent point in the research (Figure 17) [87]. 

The characterization of the compounds was performed using FT-IR and ^1^H and ^13^C NMR spectra. The determination of their structures was accomplished through the utilization of X-ray crystallography. Both of the complexes were screened in vitro for antiproliferative activity. The results showed that compound **(36)** was ineffective in T24 cells, whereas compound **(37)** had comparable cytotoxicity to that of cisplatin in the same cell line. In view of these results, complex **(37)** was also studied in vivo in sarcoma mice (SA-1). The data showed equivalent efficacy in terms of tumour growth, whilst concurrently exhibiting a substantially less pronounced impact on body weight loss when compared to that observed with cisplatin. Consequently, compound **(37)**, which can be regarded as a transplatin analogue in which two amine groups are substituted with 3-hydroxy-methyl-pyridine, has been shown to have a high antitumour effect. This is due to a different mechanism of action to those known for cisplatin [88].

It has been demonstrated that certain platinum complexes, such as *trans*-[PtCl_2_L_2_] and *trans*-[PtCl_2_(NH_3_)L] (L = planar N-donor ligand), are capable of inducing antitumour activity in cases of cisplatin-resistant tumours [28].

**Figure 18:**
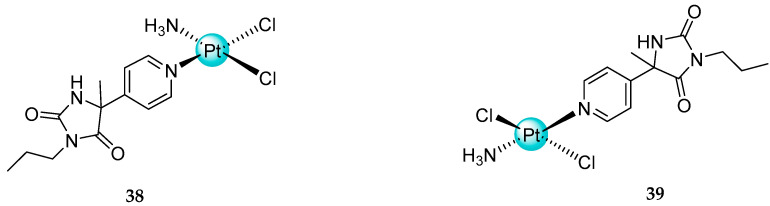
Chemical formulas of the *cis*-[PtCl_2_(NH_3_)(3-pmpy)] and *trans*-[PtCl_2_(NH_3_)(3-pmpy)].

In 2014, in our research group (Bakalova et al.), the following complexes were prepared and investigated: *cis*-dichlorido-ammine-(3-propyl-5-methyl-5(4-pyridyl)hydantoin) platinum(II) **(38)** and *trans*-dichlorido-ammine-(3-propyl-5-methyl-5(4-pyridyl)hydantoin)platinum(II) **(39)** (Figure 18) [92]. The new compounds were characterized by means of elemental analyses, IR, and ^1^H and ^13^C NMR spectroscopy. The findings of the studies demonstrated that the ligands coordinated to the platinum ion in a monodentate manner via the nitrogen atom from the pyridine ring.

The antiproliferative activity of the compounds was evaluated on four different cell lines: a human T-cell line (SKW-3), acute myeloid leukemia (HL-60), human chronic myeloid leukemia (LAMA-84), and human urinary bladder carcinoma (EJ). The study revealed that *cis*-[PtCl_2_(NH_3_)(3-pmpy)] and *trans*-[PtCl_2_(NH_3_)(3-pmpy)], where 3-pmpy is 3-propyl-5-methyl-5(4-pyridyl)hydantoin containing a single ammine group in their molecules exhibited significantly reduced cytotoxicity in comparison to the established “SAR” for di(m)mine platinum complexes.

Another approach to activate the *trans*-geometry of Pt(II) complexes is to replace the ammine ligands in cisplatin with imino ethers, which have a planar structure like pyridine and have one hydrogen atom bonded to the nitrogen, similar to amines [93,94]. For example, the complex *trans*-[PtCl_2_{(E)-HN=C(OCH_3_)CH_3_}_2_] (*trans*-EE) forms stable monofunctional adducts with DNA and is as active as cisplatin against P388 leukaemia and Lewis lung carcinoma in mice [93,95,96].

The complex *trans*-[PtCl_2_(NH_3_){(Z)-HN=C(OCH_3_)CH_3_}], which also forms stable DNA monofunctional adducts, is highly active against mouse P388 leukaemia and a xenograft of SKOV-3 human cancer cells in nude mice [97].

The *trans*-platinum complexes with iminoether as ligands have significantly higher cytotoxic activity than that of *cis*-platinum complexes. The iminoether can have either an E or Z configuration, depending upon the relative position of the alkoxy group and the N-bonded platinum atom with respect to the C=N bond [98,99]. While these *trans*-complexes react more slowly with DNA compared to cisplatin, they attain the same level of DNA binding after a 24 h period.

**Figure 19:**
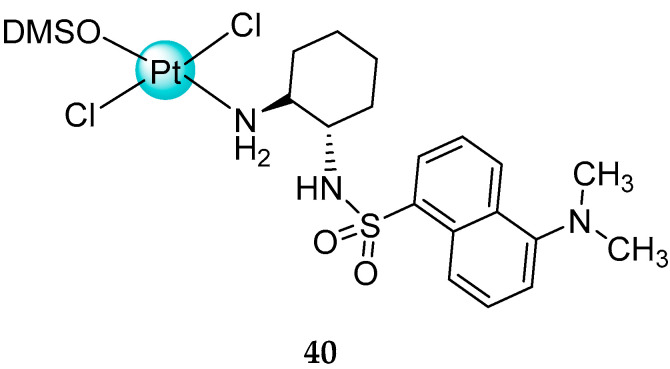
Molecular formulae of *trans*-[PtCl_2_(DMSO)L].

Novel platinum complexes with sulfonamide ligands and general formula *trans*-[PtCl_2_(DMSO)L] **(40)** (Figure 19) were synthesized and investigated for cytotoxicity in vitro on some human tumour cell lines, like melanoma (K-MEL-5), colorectal adenocarcinoma (DLD-1), luminal breast adenocarci8noma (MCF-7), testicular embryonal carcinoma (Tera-2), and cervix adenocarcinoma (HeLa). All complexes show significant cytotoxic activity [100].

The chemical structure of one of the *trans*-platinum complexes with a cyclohexyl-1,2-diamine moiety, namely dichlorido [(rac)-2-(5-(dimethylamino)-naphthalene-1-sulfonamido)cyclohexylamino] (dimethyl-sulfoxide)platinum(II) (TSPC), was proved by X-ray analysis [101]. Among the transplatin complexes tested only the latter, namely TSPC, demonstrated similar or better antiproliferative activity than cisplatin against a panel of tumour cell lines, including melanoma. The authors attributed this to the different cytotoxicity profile of the compounds from that of cisplatin. In 2017, they decided to investigate several mechanistic aspects of TSPCs in two melanoma cell lines with different TP53 statuses: SK-MEL-5 (wild-type TP53) and SK-MEL-28 (mutant TP53) [101]. This TSPC compound has been shown to have a different mechanism of action than cisplatin and can be used for co-treatment to reduce cisplatin toxicity and resistance. It can also be regarded as a promising new compound for the development of a novel cytotoxic agent and for the study of its synergism with other DNA-damaging agents [102].

As demonstrated by the literature, *trans*-[PtCl_2_L_2_] and *trans*-[PtCl_2_(NH_3_)L], where L denotes a planar N-donor ligand, have been shown to be active against cisplatin-resistant tumours [76]. In the event of one or both ammine molecules in transplatin being substituted with planar pyridine, thiazole, or non-planar piperidine and piperaxine, an increase in the cytotoxicity is observed in comparison to cisplatin and the respective *cis*-isomers [98].

For illustrative purposes, consider the findings reported by Kasparkova et al. [103,104,105]. The researchers demonstrated that substituting one ammine ligand in transplatin with a heterocyclic ligand, such as piperidine (pip) (Figure 1, piperazine, or 4-picoline, leads to the enhancement of the cytotoxic activity against human tumour cell lines that are both sensitive and resistant to cisplatin [105,106,107].

For example, *trans*-[PtCl_2_(NH_3_)(pip)], where pip is piperidine, tends to accumulate less in cells compared to transplatin. As a result, it does not significantly increase its activity in cancer cell lines [108]. It can thus be hypothesized that, there may be other biochemical factors that dominate the mechanism of action of transplatin analogues containing a heterocyclic non-leaving group such as piperidine in tumour cells. One of these factors can be associated with the modulation of the platinum–DNA interaction, or with the capacity to repair a platinum–DNA lesion [108]. Transplatin has been demonstrated not to form stable intrastrand CLs in several nucleotide sequences of double-stranded DNA, thus explaining its lack of clinical efficacy [109,110].

Kasparkova et al. in 2003 demonstrated that substituting one ammine ligand in transplatin with piperidine, piperazine, or 4-picoline enhanced the stability of 1,3-GNG intrastrand CLs in a number of sequences within short oligodeoxyribonucleotide duplexes [103]. This explains the significant cytotoxic activity of the transplatin analogues against tumour cell lines. Nevertheless, the cytotoxic effects of *trans*-[PtCl_2_(NH_3_)(pip)] and other transplatin analogues result from the replacement of the one ammine ligand in transplatin with a heterocyclic ligand [111].

**Figure 20:**
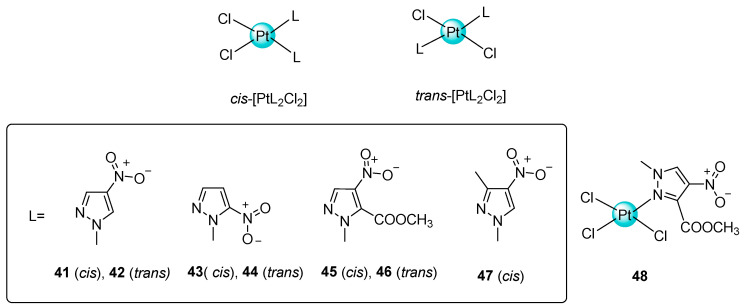
Molecular formulas of *cis*-[PtL_2_Cl_2_] and *trans*-[PtL_2_Cl_2_].

Eight novel *cis*- and *trans*-platinum(II) complexes with 1-methylnitropyrazole derivatives were synthesized and studied [81] (Figure 20).

The organic ligands are 1-methyl-3-nitropyrazole [112], 1-methyl-4-nitropyrazole [113], 1-methyl-5-nitropyrazole [111], 1,3-dimethyl-4-nitropyrazole [114], methyl 1-methyl-4-nitropyrazole-5-carboxylate [115], and methyl 1-methyl-4-nitropyrazole-3-carboxylate. The investigation established that the compound 1-methyl-3-nitropyrazole was not capable of forming a stable complex with a platinum(II) ion. The compound 1,3-dimethyl-4-nitropyrazole only formed the *cis*-isomer **(47)**, and the yield was very low. It was observed that 1-methyl-4-nitropyrazole-3-carboxylate formed only ionic complex **(48)**, with a very low yield. The compounds were tested in vitro against human lung cancer (A549), breast cancer (MCF-7), and ovarian adenocarcinoma cell lines (ES-2) in normoxic and hypoxic conditions. The results showed that compounds **(41**–**44)**, especially **(42)** and **(44)**, demonstrated superior and/or comparable activity to cisplatin on at least some cancer cell lines. The *trans*-complex with 1-methyl-4-nitropyrazole ligand (complex **(42)**) showed higher cytotoxicity than cisplatin on all human tumour cell lines tested, while the *trans*-complex with 1-methyl-5-nitropyrazole ligand (complex **(44)**) was more active than cisplatin only in the MCF-7 breast cancer line. *Cis*-complexes **(41)** and **(43)** showed similar cytotoxicity to cisplatin on the ES-2 and MCF-7 cell lines. Since the synthesized platinum complexes are only eight, the “structure–activity relationship” cannot be formulated. In this regard, the authors have tried to explain which factors influence the biological activity of the newly synthesized and studied compounds:

- The presence of a substituent in the 1-methylnitrotyrazole ring probably reduces in vitro cytotoxicity of the platinum complexes tested;
- A positive effect on the stability of the Pt complex has the maximum possible distance of the nitro group from the coordination centre of the 1-methylnitrotypyrazole ligand;
- The *trans*-isomers are more lipophilic and have a higher cytotoxic activity than the corresponding *cis*-isomers;
- Under hypoxic conditions, the tested compounds were found to be inactive or less active compared to normoxic conditions.

In order to introduce complexes with novel structures, a new class of *trans*-Pt(II)-phosphonate complexes with heterocyclic thionate ligands was investigated [116,117,118,119,120]. Further studies have shown that phosphane ligands result in increased stability of platinum complexes in the physiological environment. The increased stability would consequently lead to a reduction in required drug doses and in toxicity [120,121].

**Figure 21:**
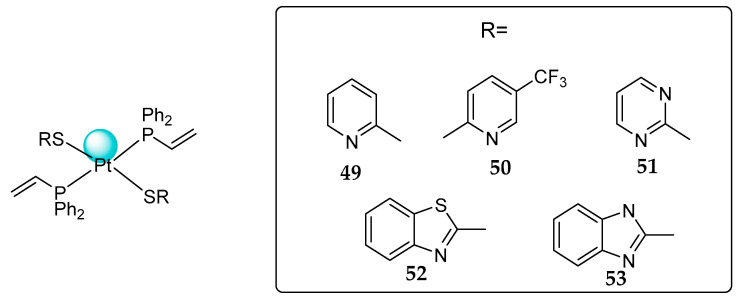
*Trans*-Pt(II) phosphane complexes with different thionate ligands.

Subsequently, in 2019, Sakamaki et al. synthesized five novel phosphane *trans*-platinum(II) complexes with the following thionate ligands: pyridine-2-thiol **(49)**; 5-(trifluoromethyl)pyridine-2-thiol **(50)**; pyrimidine-2-thiol **(51)**; benzothiazole-2-thiol **(52)**; and benzimidazole-2-thiol **(53)** [81]. The complexes were analysed using a range of analytical techniques, including elemental analyses, HR ESI-Mass, and NMR spectra. The structures of three of the complexes **(49**–**51)** were confirmed by X-ray diffraction. The X-ray data obtained showed that platinum ions coordinate with two thionate ligands *via* sulphur atoms that are in a *trans*-configuration to each other (Figure 21).

*Trans*-platinum(II) complexes were evaluated against three human tumour cell lines, lung (A549), ovarian (SKOV3), and breast (MCF-7) cancer, and showed potent cytotoxic activity. The complex **(49)** demonstrated superior cytotoxicity in comparison to cisplatin against all three human tumour cell lines. An investigation was conducted into the potential effects of **(49)** on MCF-7 cells. The results obtained demonstrated that complex **(49)** exhibited a pronounced propensity to target the genome of cancerous cells. Additionally, it has been demonstrated that complex **(49)** directly interacts with DNA, which represents its primary target. It is evident from the observations that **(49)** demonstrates the highest affinity for DNA in vitro, thus showing significant potential for future development as an antitumor agent.

A novel approach to the synthesis and study of new metal complexes with potentially antiproliferative activity involves the use of ligands, which are of significant importance in medical and biological systems [122]. For instance, flavonoid derivatives possess antioxidant, anti-inflammatory, antimicrobial and antiviral activities which renders them suitable candidates for the synthesis of new synthetic flavonoid derivatives [123,124]. Flavonoid derivatives have also been demonstrated to be effective ligands due to their cytotoxic activity to cancer cells while exhibiting minimal toxicity towards normal cells [125].

**Figure 22:**
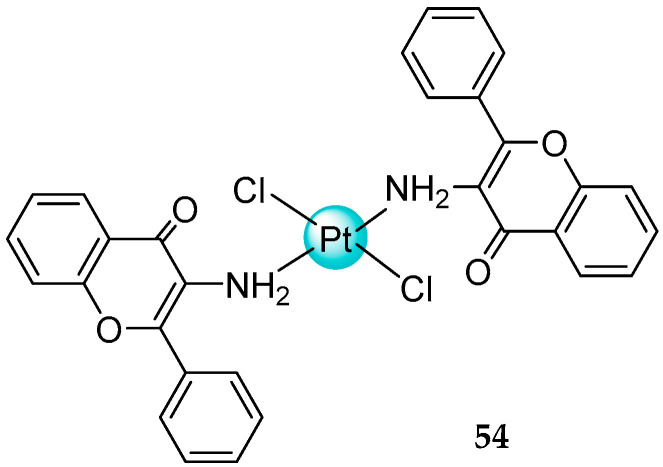
*Trans*-bis-(3-aminoflavone)dichlorido-platinum(II) complex.

A novel *trans*-bis-(3-aminoflavone)dichlorido-platinum(II) complex, designated as *trans*-[Pt(3-af)_2_Cl_2_] **(54)**, was synthesized and characterized through a range of analytical methods, including elemental analyses, ESI-MS, IR, and NMR spectroscopy (Figure 22) [122]. 

The structure of the complex was examined by X-ray analysis [122]. As demonstrated by X-ray diffraction analysis, within the complex structure, the two 3-aminoflavone ligands coordinate with the platinum ion via the nitrogen atom from the amino group. The other two ligands are chloride ions. The complex demonstrated a significant cytotoxic effect against tumour models (L1210, L1210R, HL-60, HeLa, EJ, and EJcisR) and human lymphocytes in vitro. Moreover, TCAP was found to be less toxic for normal lymphocytes than cisplatin. This property is particularly advantageous in the context of preventing potential side effects associated with drug administration.

A few years later, the same complex, *trans*-[Pt(3-af)_2_Cl_2_], was studied for its effect on the viability and cell death of two ovarian cancer cell lines, OVCAR 3 (ATCC HTB-161) and CAOV 3 (ATCC HTB-75), at doses comparable to cisplatin [76]. The investigation established that a *trans*-platinum(II) complex of 3-aminoflavone decreases the viability of ovarian cancer cells in doses comparable to those of cisplatin. Evidence has also been presented to suggest that this chemical compound can induce the expression of the proapoptotic gene, BAX (proapoptotic member of the Bcl-2 family of proteins, which plays a key role in programmed cell death).

### 2.3. Mixed Ammine/Amine Platinum Complexes

According to the previously established concept, only platinum complexes with the general formula *cis*-[PtY_2_X_2_], where Y is NH_3_/NH_2_R/NHR_2_ as carrier ligand and X is a chloride anion, 1,1-cyclobutanedicarboxylate, oxalate etc. as a leaving ligand exhibit cytotoxic activity [55]. The nature of the leaving group plays an important role in determining the side effects and cytotoxicity of platinum drugs. They can form hydrogen bonds with DNA peripheral phosphates, as non-leaving groups [25]. Furthermore, the latter could stabilize the drug–DNA adduct formed after the loss of water molecules [126,127]. The presence of chloride anions in platinum complexes leads to an increase in the cytotoxicity of the compounds, while the presence of iodide ions leads to the formation of inactive compounds. Complexes with dicarboxylate anions (e.g., carboplatin) hydrolyse slowly and form two types of complexes:

(1) Water-soluble platinum complexes, which are less toxic and show less antitumor activity;
(2) Water-insoluble platinum complexes, which have low nephrotoxicity.

**Figure 23:**
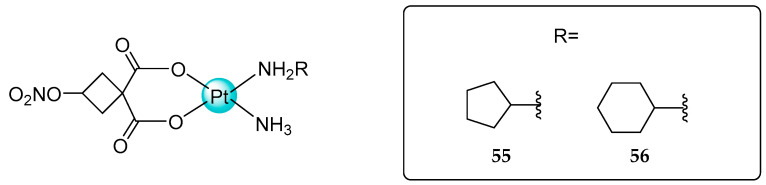
Mixed Pt(II) complexes with 3-(nitrooxy)cyclobutane-1,1-dicarboxylate ion.

The synthesis and spectral characterization of two novel platinum(II) complexes with general formula *cis*-[Pt(NH_2_R)(NH_3_)X] (where R is cyclopentylamine or cyclohexylamine and X is 3-(nitrooxy)cyclobutane-1,1-dicarboxylateach) are presented by Zhao and colleagues (Figure 23) [128].

The complexes were evaluated for cytotoxic activity in vitro on five human tumour cell lines: HepG-2 (human hepatocellular carcinoma cell line), SGC7901 (human gastric carcinoma cell line), A549 (human non-small cell lung cancer cell line), COC1 (human ovarian cancer cell line), and HCT116 (human colorectal cancer cell line). The results are expressed as IC_50_ values and then subjected to comparative analysis with those of the reference drugs cisplatin, carboplatin, and oxaliplatin. The newly synthesized Pt(II) complexes exhibited significant anticancer activity against the examined cell lines. A comparison of the results of the new compounds with those of the reference drugs cisplatin, carboplatin and oxaliplatin clearly demonstrates that the newly synthesized complexes exhibit significant antitumor activity against the cell lines tested. It is notable that compound **(55)** demonstrated higher cytotoxicity in comparison to oxaliplatin in the majority of the tumour cells examined. This observation renders compound **(55)** a promising candidate for further research as an anticancer agent.

**Figure 24:**
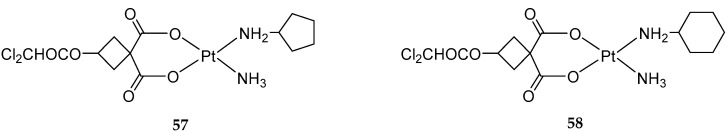
Mixed ammine/amine platinum(II) complexes with cyclopentylamine and cyclohexylamine.

The small molecule dichloroacetate (DCA) has been utilized as a treatment for patients afflicted by mitochondrial diseases, as it is capable of inducing apoptosis through selectively targeting the mitochondria of cancer cells that are resistant to anticancer drugs [129,130,131,132]. In addition, in contrast to alternative anticancer drugs, DCA does not appear to impact normal cells [133]. Liu et al. (2013) were inspired by this work and synthesized and investigated two mixed NH_3_/amine platinum(II) complexes, in which the amine component is cyclopentylamine and cyclohexylamine (See Figure 24) [134].

It has been demonstrated that both complexes exhibit significant cytotoxicity in cancer cells, selectively inducing apoptosis in such cells. Furthermore, they have been shown to have a minimal effect on normal cells. As neutral and relatively lipophilic molecules, it is hypothesized that both complexes will enter cells with greater ease, thus delivering a greater quantity of dichloroacetate into the cell in comparison to free dichloroacetate, which exists as an anion under the prevailing pH conditions of the physiological environment.

To prove that mixed ammine/amine platinum complexes have a significant cytotoxic effect, in 2016 our research group synthesized and investigated new platinum complexes with the general formula *cis*-[Pt(NH_3_)LCl_2_], where L is 3-thiolanespiro-5′-hydantoin and 4-thio-1H-tetrahydropyranespiro-5′-hydantoin. The complexes were screened by several spectroscopic methods and evaluated for cytotoxic activity on T-cell leukaemia (SKW-3) and acute myeloid leukaemia (HL-60). The data obtained were compared with those of metal-free ligands, Pt(II) complexes with the following formula, *cis*-[PtL_2_Cl_2_], and the reference drug cisplatin. The results showed that the complex *cis*-[Pt(NH_3_)LCl_2_] exhibited higher cytotoxic activity than the *cis*-[PtL_2_Cl_2_] and metal-free ligands [135].

In 2020, the same authors prepared a new platinum complex, *cis*-ammine-dichlorido-3′-methyl-tetrahydro-4H-thiopyranespiro-5′-hydantoin platinum(II) complex [136]. The obtained complex was investigated for antiproliferative activity on some human tumour cell lines, such as HL-60, REH, K-562, and colon adenocarcinoma cell line HT-29. The complex *cis*-[PtL(NH_3_)Cl_2_] proved to have a similar antitumor efficacy as that of the prototype drug cisplatin on colon adenocarcinoma model HT-29.

Two years later, the same scientific group obtained and studied a new mixed ammine/amine platinum complex, *cis*-amminedichlorido-(3′-aminethiocyclohexanespiro-5′-hydantoin)platinum(II) complex. The complex showed higher antitumor efficacy against promyelocytic leukaemia cell line (HL-60) and triple-negative breast cancer cell line (MDA-MB-231) than the complex with the same ligand but a different chemical formula, *cis*-[PtL_2_Cl_2_] [137].

A *cis*-amminechlorido-(5-methyl-5-(2-thiomethyl)ethylhydantoin) platinum(II) complex was prepared and investigated [138]. The molecular structure of the complex was investigated by X-ray analysis. The complex crystallizes in a monoclinic crystal system and a P21/c space group. The ligand coordinates bidentate to the platinum ion via a nitrogen atom from the hydantoin ring and a sulphur atom from the thioethylmethyl moiety. The molecule also contains a chloride anion and an ammonia molecule. The newly prepared complex was screened for antitumor activity on the following human tumour cell lines—K-562, HL-60, HT-29, and MDA-MB-231. It shows cytotoxic activity comparable to cisplatin on all cell lines. This could be explained by the fact that it is essentially analogous to cisplatin, which obeys classical “SARs”. It contains an ammonia molecule as the carrier ligand and a chloride ion as the leaving group. The greater lability of the Pt-S bond in the complex compared to the Pt-Cl bond in cisplatin may explain its higher cytotoxic activity observed in all cancer cell lines. The significant in vitro cytotoxicity of the complex was further investigated in an in vivo study using male and female H-albino-mice. Results showed that no signs of common toxicity had been seen.

It appears that carboxylate platinum complexes are exhibiting greater efficacy in comparison with the respective chloro analogues [139]. In a series of earlier studies, Zhang and colleagues reported on the synthesis and antitumour activity of mixed ammine/methylamine and mixed ammine/cyclohexylamine platinum(II) complexes with bidentate carboxylate ligands [26,140]. The complexes were evaluated for cytotoxic activity in vitro on some human tumour cell lines as bladder carcinoma (EJ), colon carcinoma (HCT-8), gastric carcinoma (BGC-823), immature granulocyte leukemia (HL-60), and galactophore carcinoma (MCF-7). It has been demonstrated that the complexes exhibit superior antiproliferative activity when compared to the clinically established drug cisplatin. In the case of platinum(II) complexes with amine/cyclohexylamine and dicarboxylate ligands, the presence of dicarboxylates that coordinate to platinum via oxygen atoms forms a different chelate cycle. It has been established that the size of the cycle exerts an influence on the reactivity of the leaving groups. Furthermore, evidence suggests that the size of the cycle may also have an effect on their cytotoxicity [140].

**Figure 25:**
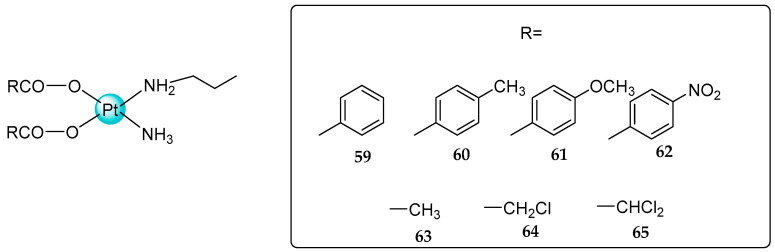
Mixed ammine/propylamine platinum(II) complexes with carboxylate ligands.

Subsequently, the same authors described the synthesis and characterization of seven new mixed ammine/propylamine platinum(II) complexes with carboxylates [141]. The prepared complexes were investigated by elemental analyses, IR, UV-Vis, and ^1^H NMR spectroscopic techniques (Figure 25).

The same human tumour cell lines were used: BGC-823, HCT-8, MCF-7, EJ, and HL-60. The cytotoxicity of the newly synthesized complexes is equal to that of cisplatin. The effects of the leaving groups on the complexes’ toxicity have been observed. It has been demonstrated that the complexes exhibit enhanced cytotoxicity when the leaving groups are aromatic carboxylates. Furthermore, the influence of substituents on benzene rings has also been demonstrated to affect the complexes’ activity. It is therefore concluded that mixed ammine/propylamine platinum(II) complexes constitute a novel class of anticancer agents and as such merit further consideration with a view to identifying lead compounds for use in the development of novel anticancer drugs [120].

**Figure 26:**
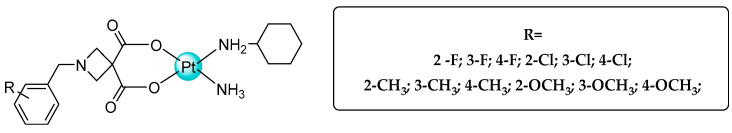
Mixed ammine/cyclohexylamine Pt(II) complexes.

A series of mixed ammine/cyclohexylamine Pt(II) complexes have been synthesized and characterized. In the complexes, the leaving groups have been modified, but the carrier groups have not (Figure 26) [142].

Some of the compounds showed cytotoxicity comparable to cisplatin and oxaliplatin and superior to carboplatin against the HepG-2, MCF-7, A549, and HCT-116 human tumour cell lines. Furthermore, the authors found that the methoxy group in the aryl ring had a more positive effect on in vitro cytotoxicity than methyl, fluorine, and chlorine substituents.

## 3. Conclusions

In recent years, there has been an increased academic interest in the synthesis and study of novel platinum complexes that exhibit higher levels of cytotoxicity and reduced side effects. It is estimated that a significant proportion of platinum complexes that are synthesized each year belong to the Pt(IV) complex compounds group. It has been established that both the axial and equatorial ligands are responsible for the increased cytotoxicity of platinum complexes. The antiproliferative activity of these compounds is explained by the reduction of Pt(IV) to Pt(II) species via reducing agents, which results in the elimination of the axial ligands. *Trans*-platinum complexes also showed significant cytotoxic activity comparable to that of the corresponding *cis*-isomers and cisplatin. This challenges the paradigm of *cis*-geometry and necessitates a re-evaluation of the SAR of antitumour platinum complexes. The nature of the leaving group is of importance in the determination of the side effects and cytotoxicity of platinum drugs. It has been established that mixed ammine/amine platinum complexes are less toxic and have equivalent or greater cytotoxic activity compared to cisplatin.
